# Association between patent foramen ovale and migraine: evidence from a resting-state fMRI study

**DOI:** 10.1007/s11682-024-00868-9

**Published:** 2024-02-21

**Authors:** Yusha Tang, Huaiqiang Sun, Chris Plummer, Simon J Vogrin, Hua Li, Yajiao Li, Lei Chen

**Affiliations:** 1 Department of Neurology, West China Hospital of Sichuan University, No. 37 Guoxue Road, Chengdu, Sichuan Province 610041 China; 2 Department of Radiology, West China Hospital, Sichuan University, Chengdu, China; 3 Department of Neuroimaging, Swinburne University of Technology, Hawthorn, VIC Australia; 4 Department of Neurosciences, St Vincent’s Hospital, Melbourne, VIC Australia; 5 Department of Cardiology, West China Hospital, Sichuan University, Chengdu, China

**Keywords:** Migraine without aura, Patent foramen ovale, Independent component analysis, Functional connectivity

## Abstract

A relationship between migraine without aura (MO) and patent foramen ovale (PFO) has been observed, but the neural basis underlying this relationship remains elusive. Utilizing independent component analysis via functional magnetic resonance imaging, we examined functional connectivity (FC) within and across networks in 146 patients with MO (75 patients with and 71 patients without PFO) and 70 healthy controls (35 patients each with and without PFO) to elucidate the individual effects of MO and PFO, as well as their interaction, on brain functional networks. The main effect of PFO manifested exclusively in the FC among the visual, auditory, default mode, dorsal attention and salience networks. Furthermore, the interaction effect between MO and PFO was discerned in brain clusters of the left frontoparietal network and lingual gyrus network, as well as the internetwork FC between the left frontoparietal network and the default mode network (DMN), the occipital pole and medial visual networks, and the dorsal attention and salience networks. Our findings suggest that the presence of a PFO shunt in patients with MO is accompanied by various FC changes within and across networks. These changes elucidate the intricate mechanisms linked to PFO-associated migraines and provide a basis for identifying novel noninvasive biomarkers.

**Supplementary Information:**

The online version contains supplementary material available at 10.1007/s11682-024-00868-9.

## Introduction

Migraine is the second most common cause of disability (Steiner et al., 2020) and affects approximately 14.1% of the global population, with two-thirds of cases being migraine without aura (MO) (Safiri et al., 2022). Although drug therapy remains the primary treatment approach, a substantial number of patients do not respond to first-line medications (Holland & Goadsby, 2018). Therefore, gaining a deeper understanding of the pathogenesis of MO is crucial for developing more effective targeted treatments. Patent foramen ovale (PFO), known as the “back door to the brain” (Ning et al., 2013), is the most common congenital intracardiac right-to-left shunt (RLS) in adults (Homma et al., 2016). In 1998, Del Sette first reported a correlation between PFO and migraine (Del Sette et al., 1998). In a previous large community-based study, we found a strong association between PFO and MO (Tang et al., 2022). However, the conclusions are controversial, and the corresponding results showed that PFO closure only completely cured migraines in some patients (Kahya Eren et al., 2015; Mojadidi et al., 2021; Takagi & Umemoto, 2016; Tariq et al., 2016). Therefore, we aimed to further explore the pathophysiological mechanisms underlying PFO and MO.

Neuroimaging techniques offer an objective means to investigate the migraine process by measuring blood oxygen level-dependent signals (Fleming & Carlsson, 2014; Schwedt & Dodick, 2009). Jia et al. (Jia, 2021) used functional magnetic resonance imaging (fMRI) to show that patients with PFO exhibit different manifestations in the temporal lobe, bilateral cerebellar hemisphere, and thalamus, all of which have been proven to be involved in the pathophysiological mechanisms of migraine. However, there are limitations that prevent the distinction of potential changes related to PFO from those associated with migraine. Hence, further research on patients with both migraine and PFO using fMRI is essential to ascertain whether PFO contributes to alterations in brain function. Additionally, brain dysfunction associated with migraines may extend beyond isolated regions and involve alterations in distributed brain networks (Lee et al., 2019). Brain network alterations can reveal high-dimensional dysfunction or reorganization in patients, which may affect the segregation and integration of information. To date, no studies have explored the effects of PFO on functional brain networks in patients with MO, and it is still unknown whether PFO affects brain functional networks differently in patients with MO than in those without migraine.

We used resting-state fMRI to capture spontaneous brain fluctuations in patients with MO and normal controls (Shi et al., 2020) and investigated abnormalities in within-network and internetwork resting-state functional connectivity (rsFC) using independent component analysis (ICA) unconstrained by a priori assumptions (Beckmann et al., 2005). The aim of this study was to elucidate the individual effects of MO and PFO, as well as their interaction, on brain functional networks and to explore the relationships between these network alterations and disease severity.

## Methods

### Participants

This study was approved by the Medical Ethics Committee of West China Hospital of Sichuan University. Written informed consent was obtained from all participants. Patients were recruited from May 2021 to January 2023 from the outpatient department of West China Hospital, and the diagnosis of MO was made according to the International Classification of Headache Disorders-III criteria (Olesen, 2018). A standardized contrast transthoracic echocardiography protocol (Silvestry et al., 2015; Tang et al., 2022) was used to assess PFO while the patients were at rest or performing Valsalva maneuvers (the diagnostic criteria and auxiliary examination procedures are shown in the Supplementary material).

Participants who met all of the following inclusion criteria were included in the study: (1) 18–50 years of age; (2) right-handed; and (3) met the criteria for migraine without aura. Patients with migraine with PFO and a grade III RLS were assigned to the MO^+^/PFO^+^ group, and those with migraine without PFO were assigned to the MO^+^/PFO^−^ group. Participants were excluded from the study if they met the following exclusion criteria: (1) had claustrophobia or any other condition incompatible with MRI, (2) had additional neurological or psychiatric disorders, (3) had alcohol or substance abuse, or (4) were pregnant or breastfeeding. MRIs of all participants were jointly inspected by a professional neurologist and an experienced neuroradiologist to exclude those with gross brain abnormalities.

Eligible patients were also instructed to maintain a headache diary for one week before and after the MRI scan. To avoid potential medication interference with BOLD signal changes, patients were instructed not to take any medication for at least 72 h before fMRI. All patients remained migraine free for 72 h before and after the MRI scan.

Age-matched healthy participants were recruited via poster advertisements from May 2021 to January 2023. The healthy controls also underwent screening using the standardized contrast transthoracic echocardiography protocol. Controls who met the following criteria underwent an MRI scan: (1) had grade III RLS (MO^−^/PFO^+^ group) and (2) lacked PFO (MO^−^/PFO^−^ group).

### Migraine assessment

Pain severity was measured by the visual analog scale (VAS), a 10-cm line positioned with anchors on the left side indicating “no pain” and on the right side indicating “pain as bad as it could possibly be”. Participants were asked to mark the line according to the severity of their worst headache pain in the preceding two weeks. Headache-related disability was accurately assessed using the Headache Impact Test-6 (HIT-6) (Houts et al., 2020).

### Imaging protocol

Images were acquired using a 3T MR scanner (Tim Trio, Siemens Healthineers) with a 32-channel head coil at the West China Hospital of Sichuan University. All the subjects were asked to lie in the supine position with a molded liner to minimize head motion. Image quality was monitored in real time. fMRI images were acquired using a multiband EP2D-BOLD sequence with the following parameters: repetition time (TR) = 700 ms, echo time (TE) = 37.8 ms, flip angle = 52°, field of view (FOV) = 210 × 177 mm^2^, slice thickness = 2.1 mm, 64 axial slices, and multiband factor = 8. fMRI data were acquired using left-right and right-left phase encoding orientations, with 415 volumes in each direction, to decrease the scan time and minimize image distortion (Smith et al., 2013). Subjects were asked to close their eyes, stay awake, and clear their minds of all thoughts during the fMRI acquisition. High-resolution T1-weighted volumes were acquired by a magnetization-prepared rapid gradient-echo sequence with the following parameters: inversion time = 1000 ms, TR = 2400 ms, TE = 2.01 ms, flip angle = 8°, 208 sagittal slices, matrix size = 320 × 320, FOV = 256 × 256 mm^2^, and voxel size = 0.8 mm isotropic. High-resolution T2-weighted volumes were acquired by a 3D turbo spin‒echo sequence with variable flip-angle echo trains, TR = 3200 ms, and TE = 565 ms. The geometric parameters were the same as those used in the T1-weighted scan protocol.

### Preprocessing

The preprocessing procedure followed the standard Human Connectome Project preprocessing pipeline and consisted of five main steps (Glasser et al., 2013). Images were corrected for distortions caused by gradient nonlinearity and distortions associated with head motion and phase encoding, underwent cross-modal registration, and were aligned with the Montreal Neurological Institute (MNI) brain space. All preprocessed images were further checked for head motion using an interframe shift (Power et al., 2012) to ensure data quality. The mean interframe shift for all subjects in both datasets was < 0.2 mm. Single-person spatial ICA with automatic dimensionality estimation was performed in FSL, followed by automatic ICA-based denoising. Linear regression was then performed on the full mixture matrix estimated by ICA to remove variance assigned to artifact components (Griffanti et al., 2014; Salimi-Khorshidi et al., 2014). The HCP_hp2000.RData dataset was used as a classification training set, and its accuracy reached 99% (Smith et al., 2013).

### Statistical analysis

The FSL tool was also used to perform spatial group-level ICA. Preprocessed, registered to MNI space and bandpass-filtered BOLD images of the entire group of participants were used as input. The resting-state network was determined by visual inspection by two experienced neurologists. Independent component maps associated with motor function, white matter, cerebrospinal fluid, or physiological noise were classified and excluded from further study. The remaining 11 networks were identified as “classical” resting-state networks (RSNs) according to previous reports (Beckmann et al., 2005; Schumacher et al., 2018; Smith et al., 2009). Anatomical locations were determined based on the Harvard–Oxford cortical and subcortical structural atlases (Desikan et al., 2006).

Dual regression (Nickerson et al., 2017) was performed with all 11 identified RSNs concatenated in a single 4D image to obtain subject-specific representations of the RSN spatial maps and associated subject-specific time courses. Group differences were tested using random permutation testing in FSL (Winkler et al., 2014) with 5000 permutations and threshold-free clustering enhancement (TFCE) (Chen et al., 2018) to correct for multiple comparisons.

The FSLNets package was used to determine the connectivity between the RSNs. Correlations were calculated between all pairs of RSNs, and the resulting correlation coefficients were converted to z scores for further analysis. The main effects of migraine and PFO, as well as the migraine * PFO interaction, were analyzed using a general linear model with age and sex as covariates for two-way analysis of variance (ANOVA). A total of 5000 permutations were performed for each comparison. The results were familywise error corrected for multiple comparisons and considered significant at the *p* < 0.05 threshold.

ANOVA and chi-square tests were used to compare the demographic and clinical characteristics of the four groups. In the migraine patient group, Spearman’s rank correlation was used to assess potential correlations between FC (mean connectivity within clusters with significant differences from dual regression and internetwork rsFC for connections with significant intergroup differences from FSLNets) and clinical scores, including the VAS score for pain intensity, HIT-6 score for disease severity, and migraine course.

## Results

### Demographics and clinical characteristics

A total of 146 patients with MO were enrolled, including 75 patients with grade III PFO and 71 patients without PFO. Seventy matched controls were included in this study, including 35 with grade III PFO and 35 without PFO. There were no significant differences in age or sex distribution among the four groups (*p* > 0.05; Table 1). Moreover, the clinical symptoms and disease course were not significantly different between the MO^+^/PFO^+^ and MO^+^/PFO^−^ groups (all *p* > 0.05). However, the HIT-6 score in the MO^+^/PFO^+^ group was significantly greater than that in the MO^+^/PFO^−^ group (*p* = 0.024).

**Table 1:** Demographic and clinical characteristics of the patients

Characteristics	MO^+^		MO^−^		P-value
	PFO^+^ (n = 75)	PFO^−^(n = 71)	PFO^+^(n = 35)	PFO^−^ (n = 35)	
Age (years)	34.51 (8.90)	35.36 (8.75)	33.28 (11.74)	31.28 (6.97)	0.215
Female	62 (82.7)	57 (80.3)	27 (77.1)	27 (77.1)	0.874
Course (years)	12.36 (8.37)	14.04 (7.86)			0.649
Frequency (number/month)	5.18 (5.99)	4.78 (5.52)			0.739
VAS	7.34 (1.41)	7.58 (1.40)			0.876
HIT-6	64.29 (6.63)	62.14 (4.56)			0.024

MO, migraine without aura; PFO, patent foramen ovale; VAS, visual analog scale; HIT-6, Headache Impact Test-6

### Identification of resting-state brain networks using ICA

Eleven components were considered to represent neuronal signals, including the left frontoparietal network (LFPN), right frontoparietal network (RFPN), occipital pole network (OPN), dorsal attention network (DAN), default mode network (DMN), salience network (SN), lingual gyrus network (LGN), medial visual network (MVN), sensorimotor network (SMN), and auditory network (AN). The DMN appeared to be divided into two subcomponents in our sample, with core features such as the precuneus and superior frontal gyrus in component 5 and the precuneus and posterior cingulate cortex in component 10 (spatial maps and locations of the 11 RSNs are shown in Fig. S1 and Table S1).

### Main effects and MO*PFO interaction effect on the within-network rsFC

**Fig. 1:**
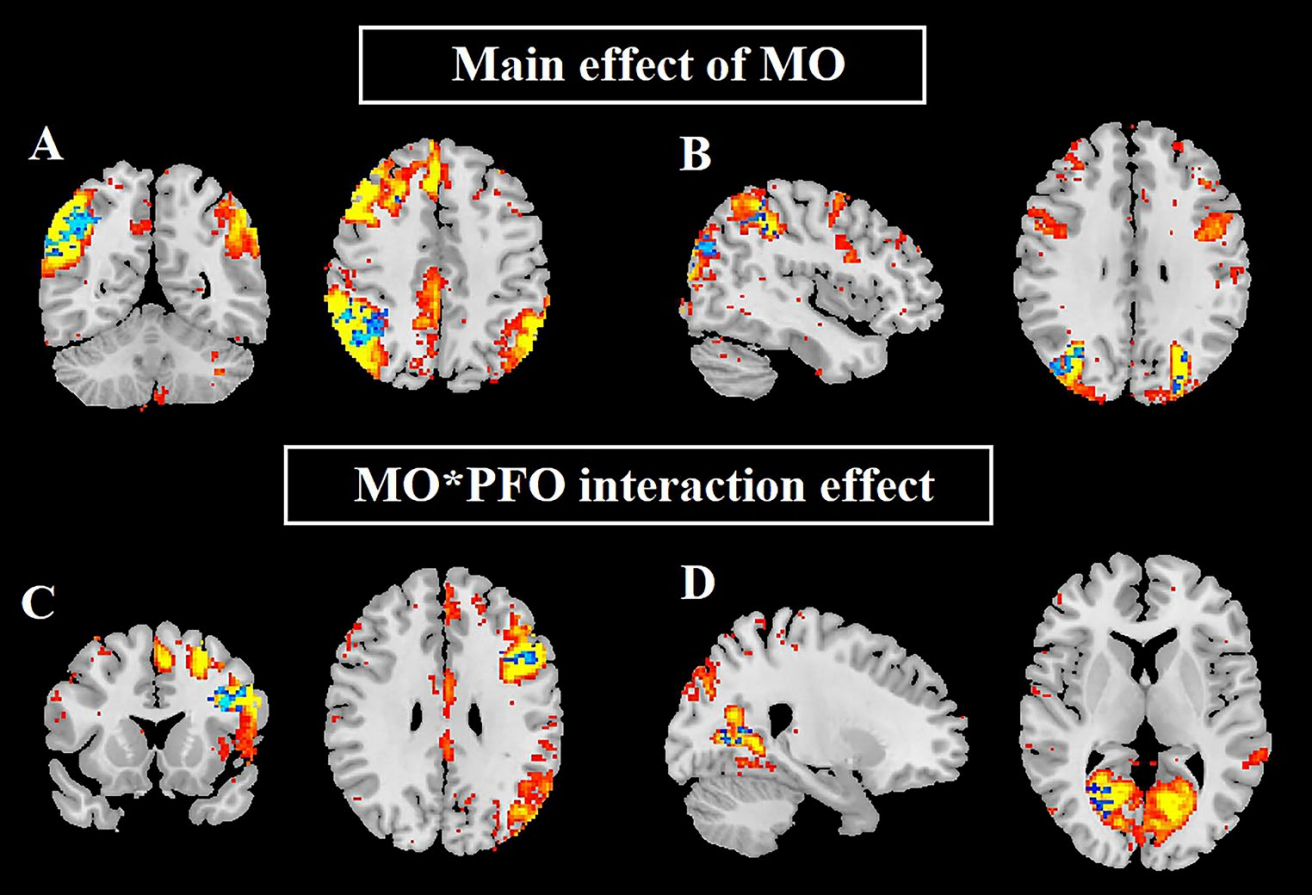
Within-network functional connectivity differences. MO, migraine without aura; PFO, patent foramen ovale. The resting network position is displayed in red and yellow. Clusters with reduced connectivity are shown in blue. (**A**) Right frontoparietal network; (**B**) dorsal attention network; (**C**) left frontoparietal network; (**D**) lingual gyrus network

The main effect of MO revealed a reduction in within-network rsFC in the RFPN between the right angular gyrus and lateral superior occipital cortex (Fig. 1A; Table 2) and the DAN between the bilateral lateral superior occipital cortex and the right superior parietal lobule (Fig. 1B; Table 2). Notably, no main effect of PFO on brain clustering was found, indicating that PFO alone did not have a significant effect on the within-network rsFC. A significant negative interaction effect between MO and PFO was observed in the LFPN, in the left middle and inferior frontal gyrus (pars triangularis) (Fig. 1C; Table 2) and in the LGN, in the bilateral intracalcarine cortex (Fig. 1D; Table 2).

**Table 2:** Within-network functional connectivity differences

Group differences	Resting-state networks	Brain areas	No. of clusters	voxels	MNI		
					X	Y	Z
MO^+^ < MO^-^	Right frontoparietal network	Right: Angular gyrus, lateral superior occipital cortex, supramarginal gyrus, and superior parietal lobule	1	424	58	-52	26
	Dorsal attention network	Bilateral: Lateral superior occipital cortex Right: Superior parietal lobule	1	244	42	-72	28
			2	32	46	-76	20
			3	30	-22	-64	34
Interaction	Left frontoparietal network	Left: Middle and inferior frontal gyrus (pars triangularis)	1	102	-36	20	26
			2	66	-54	28	2
	Lingual gyrus network	Bilateral: Intracalcarine cortex	1	62	14	-70	12
			2	53	-8	-76	14

MO, migraine without aura; TFCE correction, p < 0.05

We found no significant correlations between clinical scores and mean within-network rsFC in the migraine group (MO^+^/PFO^-^ and MO^+^/PFO^+^ groups) for the clusters that showed significant group differences.

### Main effects and MO*PFO interaction effect on the internetwork rsFC

The averaged individual correlation matrices were subjected to hierarchical clustering based on the temporal similarity of the full correlation matrices. The resulting hierarchical clustering dendrogram (Fig. S2) revealed meaningful functional grouping of the 11 networks into two distinct clusters with well-defined functional roles. The first cluster (represented by the red line) primarily consisted of the primary and secondary/higher-order visual cortex, subcomponents of the DMN, prefrontal-parietal network and attention components. The second cluster (represented by the blue line) contained the sensory cortex (components 9 and 11) and the SN.

**Fig. 2:**
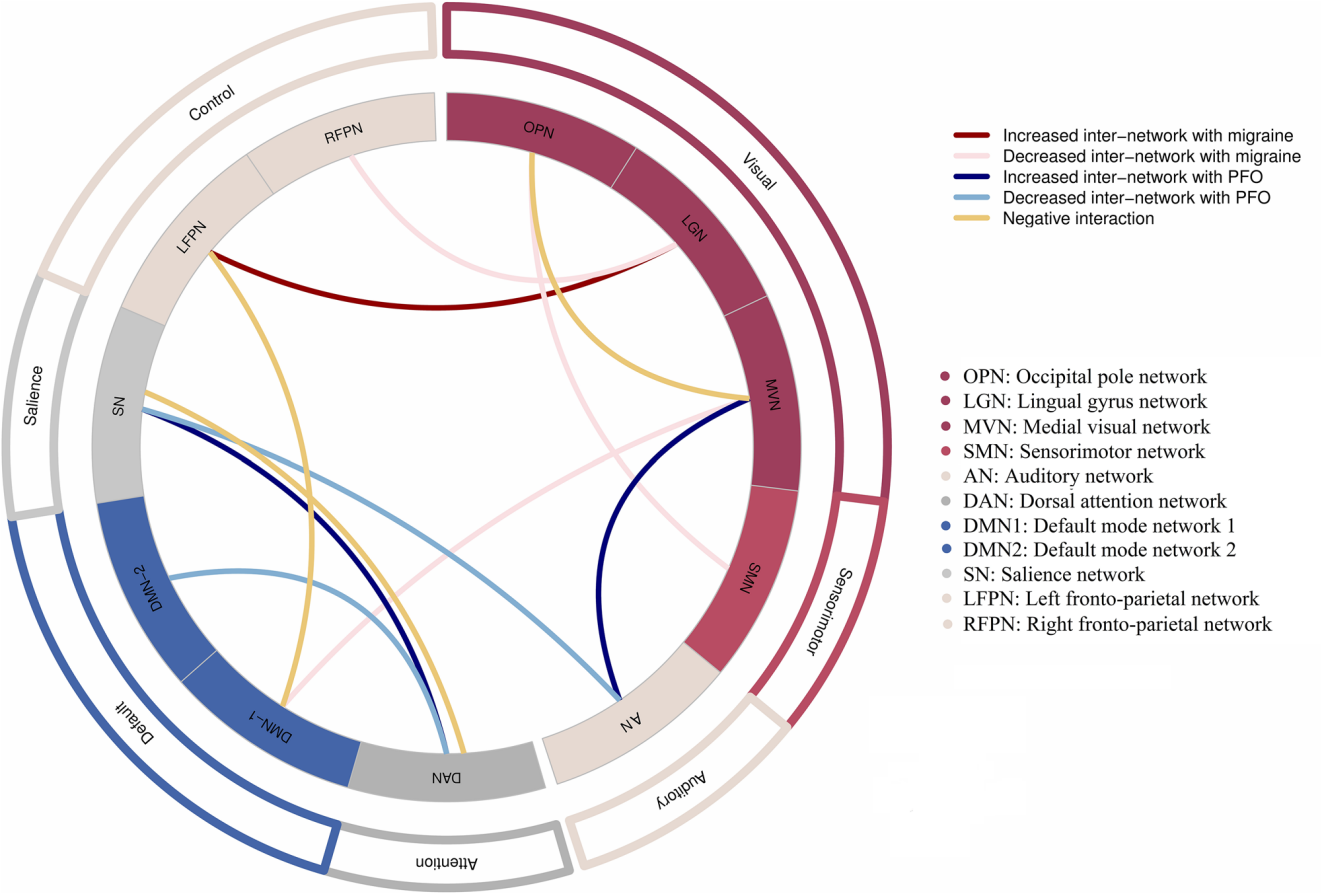
Internetwork functional connectivity differences

The main effect of MO was characterized by enhanced rsFC between the LFPN and LGN, as well as decreased rsFC between the DMN1 and MVN, the RFPN and LGN, and the OPN and SMN (*p* < 0.05; Fig. 2). Conversely, the main effect of PFO was characterized by increased rsFC between the MVN and AN and between the DAN and SN (*p* < 0.05; Fig. 2). Additionally, the main effect of PFO was characterized by decreased rsFC between the SN and AN and between the DAN and DMN2 (*p* < 0.05; Fig. 2). Notably, interactions were observed in rsFC between the LFPN and DMN1, between the OPN and MVN, and between the DAN and SN (*p* < 0.05; Fig. 2). The uncorrected results are shown in the Supplementary material (Table S2).

**Fig. 3:**
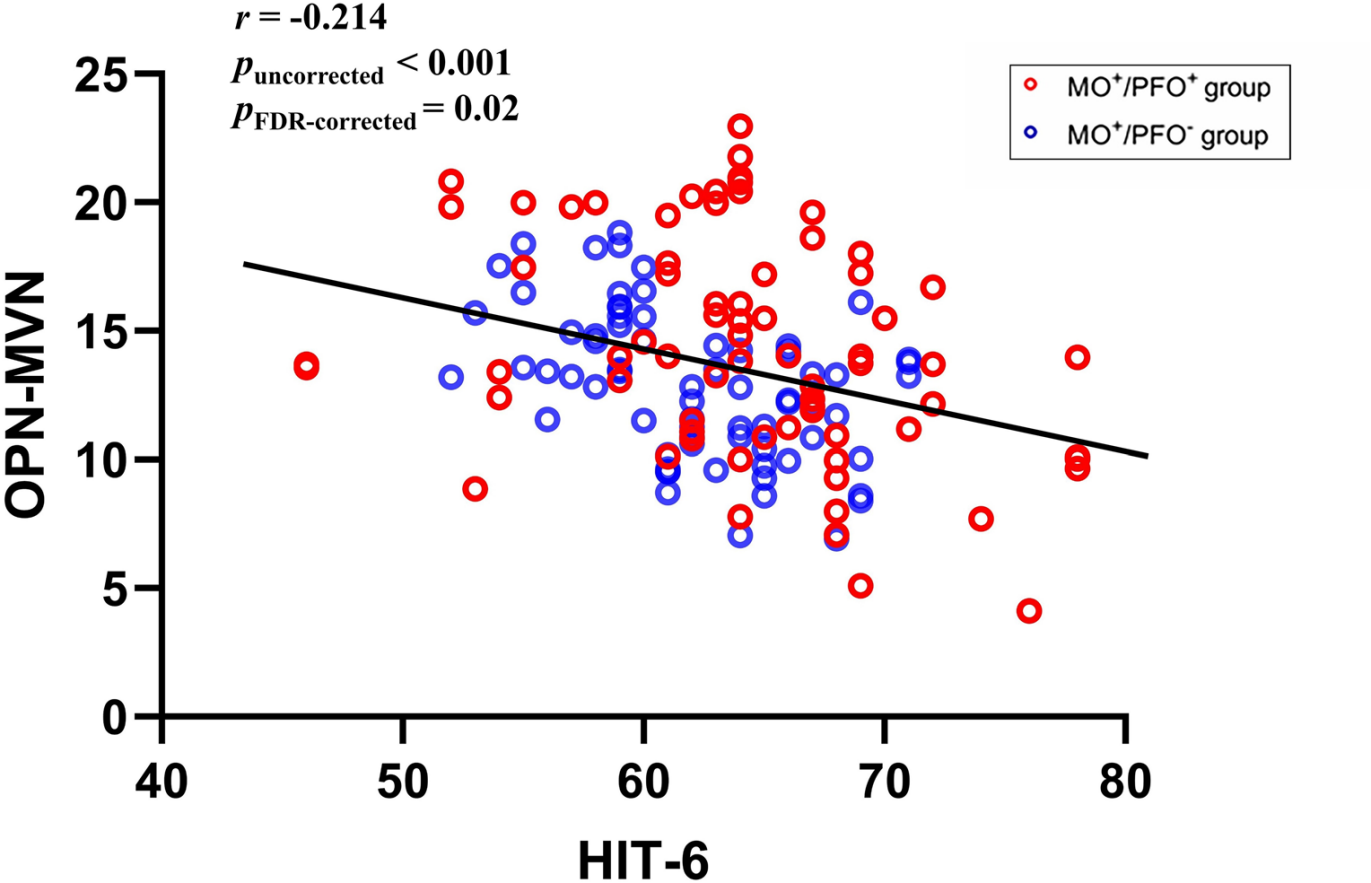
Scatter plot visual representations of the correlation analyses. MVN, medial visual network; OPN, occipital pole network; HIT-6, headache impact test-6; FDR, false discovery rate

After applying FDR correction for multiple comparisons, the rsFC between the OPN and MVN was negatively correlated with the HIT-6 score (*r* = -0.214, *P*_FDR−corrected_ = 0.02, *p*_uncorrected_ < 0.001; Fig. 3). As an additional exploratory analysis, other connectivity edge strengths that had an uncorrected p value < 0.05 are shown in Fig S3.

## Discussion

This study represents the first attempt to determine the primary effects of MO and PFO and investigate their interaction effects on brain functional networks. The key findings include the following: (1) Alterations in within-network and internetwork FC due to the main effect of MO were observed. Notably, the rsFC between the DMN1 and MVN was significantly correlated with the VAS score, and the rsFC between the RFPN and LGN was correlated with disease course. (2) The primary effect of PFO was exclusively observed in the internetwork rsFC across the visual network, auditory network, DMN, DAN, and SN. (3) The interaction effect between the MO and PFO manifested in brain clusters of the LFPN and LGN, as well as in internetwork rsFC between the LFPN and DMN, OPN and MVN, and DAN and SN. The rsFC between the OPN and MVN exhibited a negative correlation with HIT-6 scores.

While previous investigations have explored the relationship between MO and PFO, many of these studies have relied on clinical observations, case studies, and genetic epidemiology. Speculations in these studies suggest that factors such as microembolism, 5-hydroxytryptamine level, diluted blood, or genetic risk factors may converge to induce altered brain states that predispose individuals with PFO to migraines (Liboni et al., 2008; Lopez et al., 2012; Sevgi et al., 2012). However, there is limited high-quality evidence to support a link between migraine and PFO (Tariq et al., 2016), and the specific brain function changes associated with this relationship have not been reported in the literature. Therefore, our study examined the main and interaction effects of PFO on intrinsic brain activity, as reflected by rsFC, in patients with MO and healthy controls across four groups.

The RFPN plays a crucial role in cognitive control, top-down modulation, somatosensory perception, and pain processing (Zhao et al., 2021). Consistent with the findings of Russo (Russo et al., 2012), our study revealed impaired rsFC in the RFPN among patients with MO. However, Xue (Xue et al., 2012) reported greater rsFC in the RFPN of patients with MO. These divergent findings regarding RFPN rsFC changes should not be considered contradictory because we believe that they reflect the complex nature of the neural physiopathology of migraine (Pietrobon & Moskowitz, 2013).

Our results revealed altered rsFC between the FPN and LGN in patients with MO. The observed decrease in rsFC is likely a result of hyperexcitability within the pain pathway, leading to disruptions between the FPN and its associated external regions (Höffken et al., 2009). Wei highlighted the function of the lingual gyrus (LG) in pain processing and progression (Wei et al., 2022). Similarly, patients with migraine exhibit notable neural activation in the LG when exposed to negative affective stimuli (Wang et al., 2017). Additionally, the internetwork rsFC between the LGN and RFPN was negatively correlated with disease course in patients with MO, which suggested that pain-related brain regions exhibit less connection to the RFPN as the disease progresses. Interestingly, we observed a significant interaction effect between MO and PFO within the LGN. Conversely, the LG plays a crucial role in the cortical spreading depression (CSD) hypothesis, which proposes that CSD contributes to the initiation and persistence of migraine through trigeminal vascular system activation (Dahlem & Hadjikhani, 2009). This study may explain the role of CSD in the pathogenesis of PFO-associated migraine, providing a conceptualization of PFO-associated migraine as a dysfunction of brain networks.

Consistent with the findings of previous studies (Soheili-Nezhad et al., 2019), we confirmed that migraine patients displayed reduced rsFC between the visual cortex and the DMN and SMN. We first demonstrated the effect of the interaction between PFO and MO on rsFC between different visual subnetworks. Aberrant feedback projections to the visual network may exacerbate migraine distress and may be involved in migraine pathophysiology (Cui et al., 2021; Wei et al., 2019). The visual cortex is the primary site of hypoperfusion and CSD onset (Woods et al., 1994), both of which are implicated in the pathophysiological mechanism of migraine. Given the crucial role of the visual cortex, these findings support our findings and suggest that intravisual microcircuit abnormalities are essential in the potential mechanisms of PFO-associated migraines.

The distraction strategy, a natural response to avoid suffering, is related to specific functional activities between specific brain networks. We observed an interaction between PFO and MO across these networks. The DMN is involved in self-referential processing and mind-wandering (Buckner et al., 2008; Mason et al., 2007), whereas the LFPN is essential for attention. FC between these networks demonstrates the brain’s self-compensatory adaptation and coping responses to persistent migraine attacks (Borsook et al., 2012). Similarly, the DAN is crucial for maintaining stable attentional states (Tamber-Rosenau et al., 2018) and aids in selecting relevant stimuli on which to focus attention when collaborating with the SN. Our findings partially support previous research demonstrating that headache-related impact and disability are significantly greater among migraine patients with moderate or large RLS than among non-RLS patients (He et al., 2020) and reveal the essential role of dysfunctional brain networks in the potential mechanisms associated with PFO and clinical features. Attending to pain intensifies suffering, whereas distraction from pain results in a less painful perception. However, additional direct evidence is still needed to validate these findings.

Visual and auditory discomforts are common complaints among patients with migraine and are often linked to increased rsFC between the visual and auditory networks (Wei et al., 2022). This disruption may result in impaired multisensory processing and integration. However, the direct relationship between PFO and photosensitivity has not been explored previously. We found that the FC strength between the MVN and AN was significantly greater in individuals with PFO than in those without PFO, which extends our previous findings that RLS is independently associated with photosensitivity and might exacerbate photophobia (Dong et al., 2023). This finding suggested disrupted system-level control of the visual and auditory circuits in individuals with PFO even before they experienced headache symptoms.

This study has certain limitations. First, although contrast transthoracic echocardiography has high sensitivity and specificity for detecting PFO, it may not be suitable for screening all patients, especially those with a small RLS (Ren et al., 2013). Hence, we included only patients with large shunts in the MO^+^/PFO^+^ and MO^−^/PFO^+^ groups to avoid missing small shunts or misclassifying patients as PFO^−^ instead of PFO^+^. Second, because contrast transthoracic echocardiography is an invasive examination, the sample size of healthy participants was relatively small.

## Conclusions

Our study provides insights into the interaction between PFO and MO on brain networks and identifies a possible mediation pathway through which PFO impacts MO through within-network and internetwork FC dysfunction. This contributes to our understanding of the intricate mechanisms underlying PFO-associated migraine and lays the groundwork for identifying novel noninvasive biomarkers.

### Electronic supplementary material

Below is the link to the electronic supplementary material.

Supplementary Material 1

## Data Availability

All relevant data are within the article. Requests for anonymized data should be sent to the corresponding author on reasonable request.
