# Supplementary material for: Association between patent foramen ovale and migraine: evidence from a resting-state fMRI study
Source: Brain Imaging Behav. 2024 Feb 21;18(4):720–9. doi: 10.1007/s11682-024-00868-9 (PMC11364569; doi:10.1007/s11682-024-00868-9)
Supplement: Supplementary file 1 — Supplementary Material 1 [file 11682_2024_868_MOESM1_ESM.pdf]

Supplement to:

Association between patent foramen ovale and migraine: evidence from a resting-state fMRI study

Supplementary Methods

Diagnostic criteria and procedures of patent foramen ovale

Supplementary Results

Table S1 Locations of RSN spatial maps.

Fig S1 Spatial maps of the 11 resting-state networks.

Fig S2 Functional grouping of the 11 neural networks.

Fig S3 Correlation analyses in the migraine group.

Methods

Diagnostic criteria and procedures of patent foramen ovale (PFO)

Contrast transthoracic echocardiography was performed by 2 experienced sonographers to identify PFO using a Philips IE 33 with 1-5 MHz or 3-8MHz multiplane transducers. Subjects were assessed for PFO at rest, during a Valsalva manoeuvre (expiratory pressure at 60mm Hg measured by manometer), and coughing. A microbubble bolus from agitated solution of 8 milliliters of saline, 1 milliliter of blood, and 1 milliliter of air was injected into antecubital veins for increased sensitivity. PFO was diagnosed if microbubbles were seen in the left chambers within 3 cardiac cycles from maximum right atrial opacification and provocative maneuvers, PFO was diagnosed. The degree of RLS was quantified based on the maximum value of detected microbubbles per frame in the left atrium (during the first 3 cardiac cycles after right atrial opacification) at rest or during provocative manoeuvres: grade I indicating 1-10 microbubbles, grade II indicating 11-30 microbubbles, and grade III indicating more than 30 microbubbles or the left atrium being filled with microbubbles.

Table S1 Locations of RSN spatial maps

RSN name			Brain regions
IC-1	Left fronto-parietal network	LFPN	Left superior lateral occipital cortex, right angular gyrus, left middle frontal gyrus
IC-2	Right fronto-parietal network	RFPN	Right superior lateral occipital cortex, right angular gyrus, right middle frontal gyrus, left superior lateral occipital cortex
IC-3	Occipital pole network	OPN	Occipital Pole
IC-4	Dorsal attention network	DAN	Superior parietal lobule, supramarginal gyrus, superior lateral occipital cortex
IC-5	Default mode network 1	DMN1	Precuneus, posterior cingulate cortex, frontal Pole, superior frontal gyrus, angular gyrus, superior lateral occipital cortex,
IC-6	Salience network	SN	Insular cortex, anterior cingulate cortex
IC-7	Lingual gyrus network	LGN	Lingual gyrus, cuneal Cortex, intracalcarine cortex
IC-8	Medial visual network	MVN	Intracalcarine cortex, supracalcarine cortex, lingual gyrus, occipital pole
IC-9	Sensorimotor network	SMN	Pre- and postcentral gyrus, supplementary motor cortex
IC-10	Default mode network 2	DMN2	Precuneus, posterior cingulate cortex, angular gyrus
IC-11	Auditory network	AN	Superior temporal gyrus, planum temporale, Heschl's gyrus

RSN, resting-state networks

Anatomical locations were based on Harvard-Oxford cortical and subcortical structural atlases.

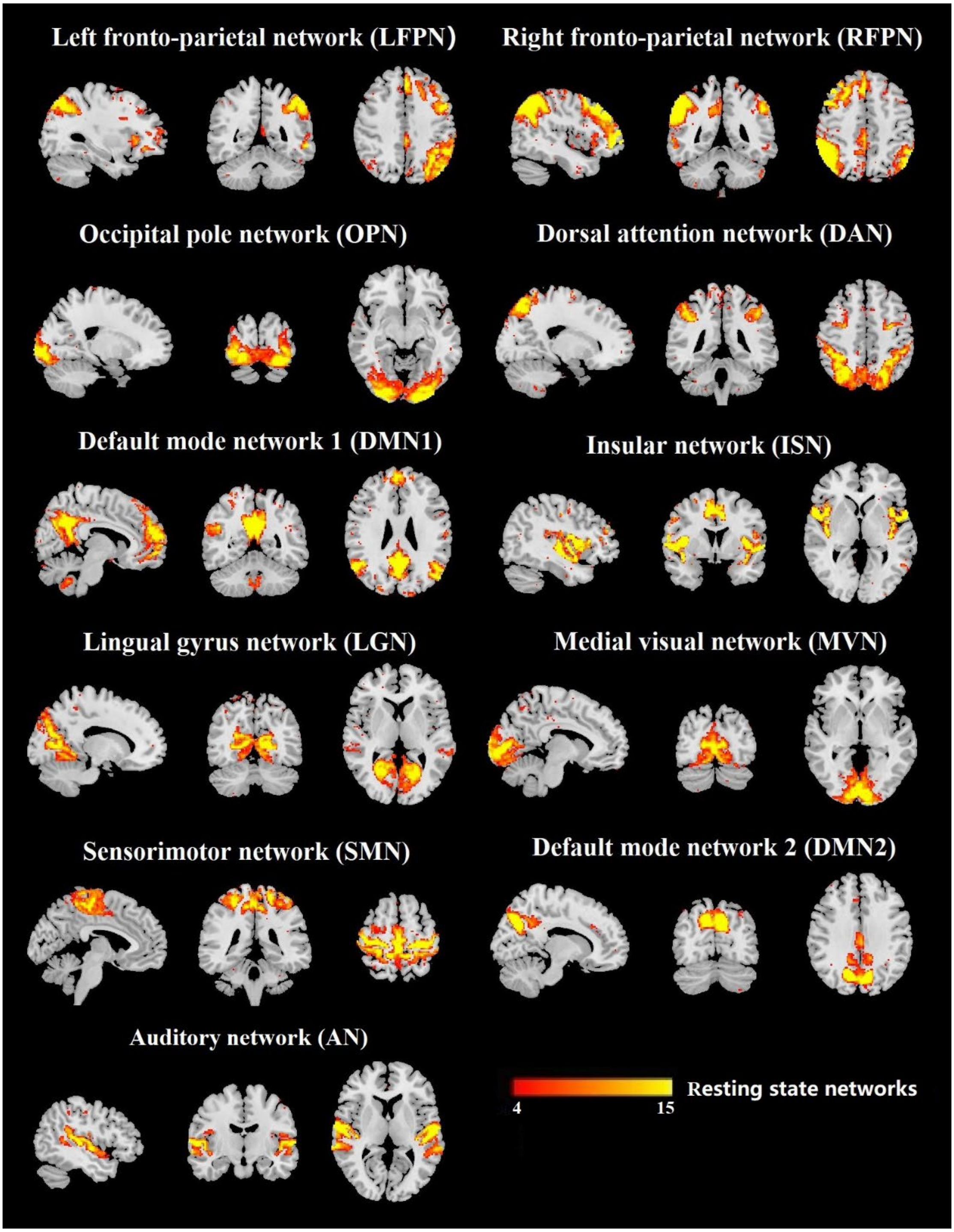

Fig S1 Spatial maps of the 11 resting-state networks. The resting network position is displayed in red and yellow.

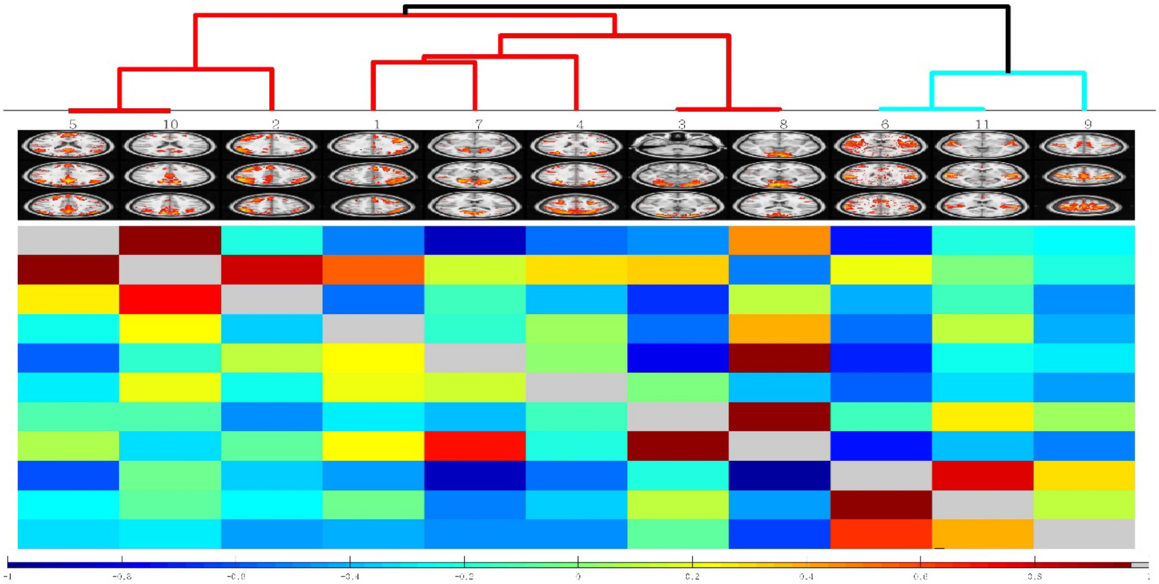

Fig S2 Functional grouping of the 11 neural networks. Z statistics for the full correlation is shown below the diagonal and for the Ridge regression (with rho = 0.1) is shown above diagonal. The resulting nodes are clustered hierarchically by using the information about temporal similarity of the full correlation matrices. The color bar represents the correlation coefficient.

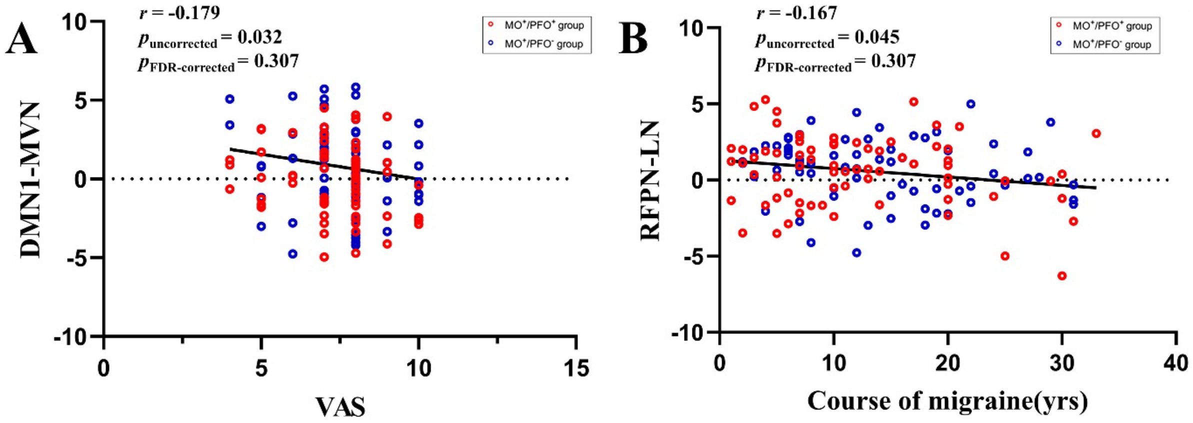

Fig S3 Correlation analyses in the migraine group. All edges are shown that have an uncorrected p-value<0.05. DMN, default mode network; MVN, medial visual network; RFPN, right frontoparietal network; LN, lingual gyrus network; VAS, visual analogue scale.
